# Natal teeth: report of two cases

**DOI:** 10.11604/pamj.2020.36.312.24861

**Published:** 2020-08-20

**Authors:** Salma Aboulouidad, Zakaria Aziz, Mohammed El Bouihi, Saad Fawzi, Abdeljalil Abouchadi, Nadia Mansouri Hattab

**Affiliations:** 1 Maxillofacial Surgery Department, University Hospital Center Mohammed VI, Marrakech, Morocco,; 2 Maxillofacial Surgery Department Avicenne Military Hospital, University Hospital Center Mohammed VI, Marrakech, Morocco

**Keywords:** Natal tooth, extraction, case report

## Abstract

Presence of teeth at birth or within a month post-delivery is a rare condition. We report here 2 cases to highlight their clinical features and discuss their possible treatment. A 7- days old female newborn with two mandibular central incisors, noticed by the parents at birth, with complaint of continuous crying, inability to suck milk and causing discomfort to the mother. The left incisor was highly mobile justifying its extraction. The second case was a female newborn referred 2 hours after delivery, for a brownish highly mobile mandibular incisor noticed by the pediatrician. Extraction was immediately made to avoid the danger of aspiration. This phenomenon can lead to complications either local such as pain on suckling or general such as undernutrition or asphyxia by aspiration. The decision to maintain or remove these teeth should be assessed in each case independently, based on degree of mobility and interference with breastfeeding.

## Introduction

Natal teeth are a rare condition referring to teeth present at birth or erupting during first month of life, its prevalence varies from a study to another, ranging from 1: 716 to 1: 30 000 [1]. It has been a subject of curiosity and study since ancient times, at first surrounded by beliefs and misconceptions like bringing misfortune in some African tribes who murdered children born with teeth, while considered as a sign of splendid future in other cultures [2]. Family of Chinese children believed that when these natal teeth would start to bite one of the parents would die. In England, the belief was that babies born with teeth would grow to be famous soldiers, whereas in France and Italy the belief was that this condition would guarantee the conquest of the world.

Historical figures such as Zoroaster, Hannibal, Luis XIV, Mazarin, Richelieu, Mirabeau, Richard III, and Napoleon may also have been favored by the presence of natal teeth [1,3]. The etiology is still debated but the most accepted theory is a superficial localization of dental follicles probably related to hereditary factors [4]. This phenomenon can lead to complications either local such as pain on suckling or general such as undernutrition or asphyxia by aspiration [5]. The purpose of this paper is to report two cases in order to present a rare disorder of dental eruption.

## Patient and observation

**Figure 1:**
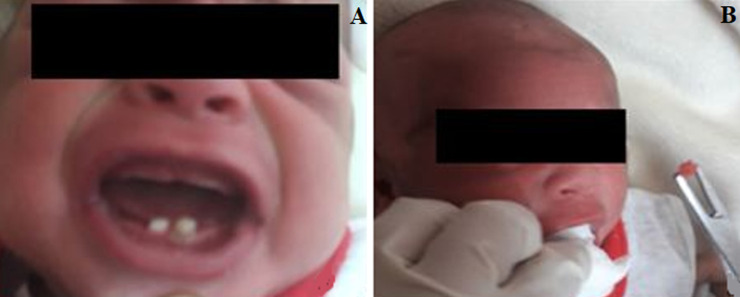
A) intraoral photograph of 7-days old female infant showing two natal mandibular incisors; B) extraction of natal highly mobile incisor

**Case 1:** a 7- days old female newborn was referred to the maxillofacial surgery department of Mohamed VI University hospital at Marrakesh for two mandibular central incisors noticed by the parents at birth, with complaint of continuous crying, inability to suck milk and causing discomfort to the mother. The infant was non syndromic and oral examination revealed two crowns of the teeth in the mandibular anterior region (Figure 1A). They were normal sized and whitish without injury of ventral face of the tongue. The left incisor was highly mobile justifying its extraction under local anesthesia to avoid aspiration, followed by a gentle curettage to remove any odontogenic remnants (Figure 1B). Before extraction, parents consent was taken and they were informed about the chances of the absence of permanent central incisor in the future, as it was difficult to perform radiographic examination to rule out whether the natal tooth belongs to permanent dentition or was supernumerary. The removed tooth had a crown but was lacking root. The baby was reevaluated seven days later and the healing seemed to be uneventful.

**Figure 2:**
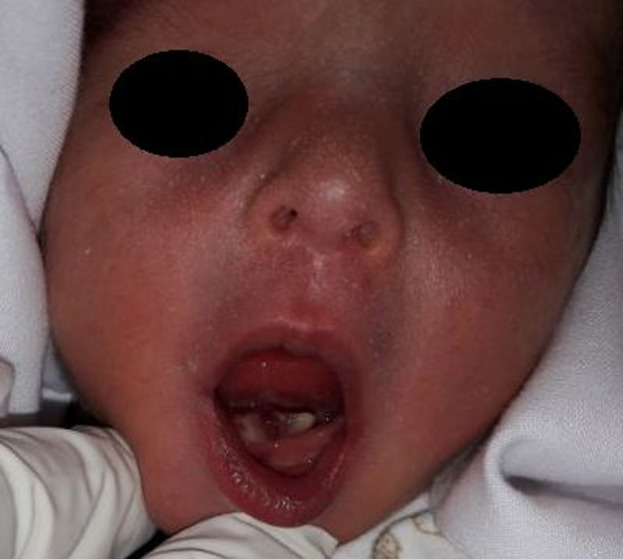
two hours old female newborn with a brownish natal mandibular incisor

**Case 2:** a female newborn referred 2 hours after an eutocic delivery to the maxillofacial surgery department of Mohamed VI University hospital at Marrakesh, for a brownish highly mobile mandibular incisor noticed by the pediatrician (Figure 2). There was a danger of aspiration of these tooth, due to which decision to extract it immediately was made. The tooth was removed under local anesthesia with an alveolar curettage. The baby was given intramuscular Vitamin K. The removed tooth had a shell crown with no root. 48 hours later, no hemorrhage sign was found.

## Discussion

Natal and neonatal teeth were first documented by Titus Livius in 59 BC. He considered natal teeth to be prediction of disastrous events. Several terms have been used in the literature to designate teeth that erupt before the normal time, such as congenital teeth, fetal teeth, predecidual teeth, dentitia praecox and dens connatalis. Currently we adopt the terminologies used by Massler and Savara who termed all teeth that are present at birth as natal teeth and those that erupt during the neonatal period (first 30 days of life) as neonatal teeth [4, 6, 7]. Many authors tried to classify this pathology on the basis of clinical characteristics such as Spoug and Feasby (1966) who suggested a classification according to their degree of maturity [8] or Hebling (1997) who recommended a 4 clinical categories classification [9]. Natal teeth are more often encountred than neonatal with a ratio 3: 1 [3, 10], and a greater predilection of female gender which is consistent with our report. The etiology of natal and neonatal teeth is still unknown. There are various hypothetical factors reported in literature by investigators which include the superficial position of tooth germ associated with hereditary factor, poor maternal health, and nutritional deficiency, maternal exposure to environmental toxins, endocrine disturbances, infections, and febrile episodes during pregnancy causing accelerated eruption. There are no studies available that confirm a causal relationship with any of the theories proposed thus far. However, the first one seems to be the most accepted possibility [2, 4].

The most common localization is the mandibular region of central incisors (85%) followed by 11% in the maxillary incisor region, 3% in the mandibular canine and molar region, and 1% in the maxillary canine and molar region. In 61% of cases, the teeth are double or in pairs and mostly correspond to normal primary dentition in 95% cases, while 5% are supernumerary [2, 4]. Clinically, the natal teeth; are small, or of normal size, conical/or of normal shape. They may reveal an immature appearance with enamel hypoplasia and small root formation. Natal teeth may exhibit a brown-yellowish/whitish opaque color [4]. The treatment plan is based on several factors: implantation and degree of mobility, interference with breast feeding and sucking, possibility of traumatic injury, and whether the tooth is part of the normal dentition or is supernumerary [2]. A clinical and radiographic examination is crucial to take the decision of removing or maintainig the tooth. If it´s diagnosed as a tooth of the normal dentition, the maintenance of these teeth in the mouth is the first treatment option, unless this would cause injury to the baby (Riga-Fede- Disease), interfere with feeding or if it´s highly mobile, with the risk of aspiration. When the tooth is well implanted, tongue ulcer can be avoided by smoothing the incisal margin [1] or covering it by composite resin. If the treatment option is extraction, these teeth can be removed with a forceps or even with the finger, with attention paid to hemorrhage risk prevented by vitamin K administration [3].

## Conclusion

Natal and neonatal teeth are rare occurrences in the oral cavity and proper evaluation and diagnosis are crucial to provide the best treatment option. The decision to maintain or remove these teeth should be assessed in each case independently, based on degree of mobility and interference with breastfeeding.
